# The Seasonal Flux and Fate of Dissolved Organic Carbon Through Bacterioplankton in the Western North Atlantic

**DOI:** 10.3389/fmicb.2021.669883

**Published:** 2021-06-18

**Authors:** Nicholas Baetge, Michael J. Behrenfeld, James Fox, Kimberly H. Halsey, Kristina D. A. Mojica, Anai Novoa, Brandon M. Stephens, Craig A. Carlson

**Affiliations:** ^1^Department of Ecology, Evolution and Marine Biology, Marine Science Institute, University of California, Santa Barbara, Santa Barbara, CA, United States; ^2^Department of Botany and Plant Pathology, Oregon State University, Corvallis, OR, United States; ^3^Department of Microbiology, Oregon State University, Corvallis, OR, United States; ^4^Division of Marine Science, School of Ocean Science and Engineering, The University of Southern Mississippi, John C. Stennis Space Center, Hattiesburg, MS, United States; ^5^Scripps Institution of Oceanography, University of California, San Diego, La Jolla, CA, United States

**Keywords:** bioavailability, bacterioplankton carbon demand, dissolved organic carbon, NAAMES, biological carbon pump

## Abstract

The oceans teem with heterotrophic bacterioplankton that play an appreciable role in the uptake of dissolved organic carbon (DOC) derived from phytoplankton net primary production (NPP). As such, bacterioplankton carbon demand (BCD), or gross heterotrophic production, represents a major carbon pathway that influences the seasonal accumulation of DOC in the surface ocean and, subsequently, the potential vertical or horizontal export of seasonally accumulated DOC. Here, we examine the contributions of bacterioplankton and DOM to ecological and biogeochemical carbon flow pathways, including those of the microbial loop and the biological carbon pump, in the Western North Atlantic Ocean (∼39–54°N along ∼40°W) over a composite annual phytoplankton bloom cycle. Combining field observations with data collected from corresponding DOC remineralization experiments, we estimate the efficiency at which bacterioplankton utilize DOC, demonstrate seasonality in the fraction of NPP that supports BCD, and provide evidence for shifts in the bioavailability and persistence of the seasonally accumulated DOC. Our results indicate that while the portion of DOC flux through bacterioplankton relative to NPP increased as seasons transitioned from high to low productivity, there was a fraction of the DOM production that accumulated and persisted. This persistent DOM is potentially an important pool of organic carbon available for export to the deep ocean via convective mixing, thus representing an important export term of the biological carbon pump.

## Introduction

Phytoplankton are prolific in the world’s oceans and are recognized to be a critical source of fresh organic matter for marine food webs and subsequently play a key role in the biogeochemical cycling of elements. Despite representing less than 0.2% of Earth’s photosynthetic biomass, marine phytoplankton have rapid turnover times and consequently are responsible for nearly half of the planet’s annual net primary production (NPP), consuming CO_2_ and elemental nutrients while generating oxygen and new organic matter (Field et al., 1998). The organic matter that is produced is partitioned as particulate organic matter (POM) and dissolved organic matter (DOM). This partitioning has a profound impact on the fate and contribution of organic matter to the biological carbon pump.

The biological carbon pump represents a combination of processes that spatially separate organic matter (particulate and dissolved) production from its remineralization (Passow and Carlson, 2012; Boyd et al., 2019), including the passive sinking flux of POM (McCave, 1975), physical deep mixing of DOM (Copin-Montégut and Avril, 1993; Carlson et al., 1994), or suspended POM (Dall’Olmo et al., 2016; Lacour et al., 2019), and zooplankton-mediated transport by vertical migration (Steinberg et al., 2000). These three export pathways can transport organic carbon to depths where a portion remains sequestered from the atmosphere for decades to centuries (Ducklow et al., 2001b).

Food web processes that control the production of DOM include direct extracellular release by phytoplankton, viral-induced or autolysis of phytoplankton cells, grazing activity (i.e., sloppy feeding, excretion, and egestion by microzooplankton grazers), and solubilization of organic particles (see review in Carlson and Hansell, 2015). Heterotrophic bacterioplankton production (BP) is the primary conduit for the uptake of bioavailable DOM and its passage to higher trophic levels or remineralization, processes key to defining the microbial loop as an important carbon-flow pathway that can modify the ocean carbon cycle (Azam et al., 1983; Azam, 1998).

The cumulative organic carbon flux through heterotrophic bacterioplankton, or gross BP [also termed bacterioplankton carbon demand (BCD)], can be estimated as the sum of net BP and the carbon that is respired as CO_2_ (Del Giorgio and Cole, 1998; Ducklow et al., 2000; Carlson and Hansell, 2015). Comparing BCD to NPP provides a useful index for evaluating the degree to which NPP can support BCD (BCD:NPP) (Cole et al., 1988). Reported values of BCD:NPP have been as low as 0 (Pomeroy and Deibel, 1986; Pomeroy and Wiebe, 2001) and have also exceeded 1 (Duarte and Agustí, 1998; Hoppe et al., 2002). The BCD:NPP ratio is an important index that can be used to identify regions of net heterotrophy, where the net out-gassing of CO_2_ can occur (Hoppe et al., 2002), and it can also serve as a harbinger of dissolved organic carbon (DOC) accumulation on diel to seasonal time scales. For instance, the enhanced primary production during a phytoplankton bloom in the Antarctic Ross sea was matched by an increase in BCD, which limited DOC accumulation during the phase of the bloom when phytoplankton division outpaced loss rates (Carlson and Hansell, 2003). Conversely, over three bloom seasons in the Sargasso sea, nearly half of the seasonally produced DOC escaped rapid microbial degradation (i.e., low BCD:NPP), resulting in DOC accumulation (Carlson et al., 1998).

The condition where BCD:NPP is low (i.e., DOC consumption is unable to match the rate of DOC release) has been termed the “malfunctioning microbial loop” (Thingstad et al., 1997). This “malfunctioning” can arise for a number of reasons. It can result from phytoplankton growth exceeding the metabolic capacity of heterotrophic consumption (Ducklow et al., 1993), the production of recalcitrant compounds (Aluwihare et al., 1997) or precursors to recalcitrant compounds (Arakawa et al., 2017), or the inability of a bacterioplankton assemblage to grow on specific types of organic matter due to community composition or gene expression (Teeling et al., 2012). It can also result from bacterioplankton–phytoplankton competition leading to nutrient limitation on BCD (Zweifel et al., 1995; Cotner et al., 1997; Church, 2008) or from predation limiting BP (Thingstad et al., 1997). It is also possible that BCD may more closely track grazer or viral-mediated release of DOM instead of instantaneous phytoplankton production (Kirchman et al., 1994). Lastly, some BCD may be supported by volatile organic compounds which are not captured in estimates of NPP measured by contemporary methods (Davie-Martin et al., 2020; Moore et al., 2020). A disconnect between BCD and NPP can arise from the combined effects of any or all of these mechanisms and can lead to the seasonal accumulation of DOC in the surface waters, i.e., the accumulation of DOC in the euphotic zone that is greater than the annual minimum concentration observed during deep winter mixing. If the fraction of accumulated DOC that is produced as, or transformed into, recalcitrant compounds persists long enough to be vertically exported to depth during the next winter’s convective mixing event, it can represent an important pathway of the biological carbon pump in systems that experience deep convective mixing or subduction (Carlson et al., 1994).

The Western North Atlantic Ocean is a region characterized by both massive seasonal phytoplankton blooms (Behrenfeld, 2010) and deep convective overturning events that can physically deliver dissolved and suspended organic matter to depth (Carlson et al., 1994; Dall’Olmo et al., 2016; Baetge et al., 2020). The bloom conditions in the Western North Atlantic provide an ideal system to explore the cumulative influence of microbial processes on the accumulation, bioavailability, and persistence of DOC, and ultimately, on the biogeochemical role of DOC in the biological carbon pump. Here we present data collected over a seasonal cycle as a part of the NASA North Atlantic Aerosols and Marine Ecosystems Study (NAAMES). We combined observations from *in situ* bacterioplankton measurements and experimental DOC remineralization experiments with estimates of net community production (NCP) partitioned as DOC (Baetge et al., 2020): (1) examine the efficiency at which bacterioplankton use DOC, (2) examine seasonal variability in the fraction of NPP supporting BCD, (3) assess the bioavailability of the seasonally accumulated DOC, and (4) evaluate how export potential of seasonally accumulated DOC varies over a bloom cycle in the Western North Atlantic.

## Materials and Methods

### Study Region

**FIGURE 1:**
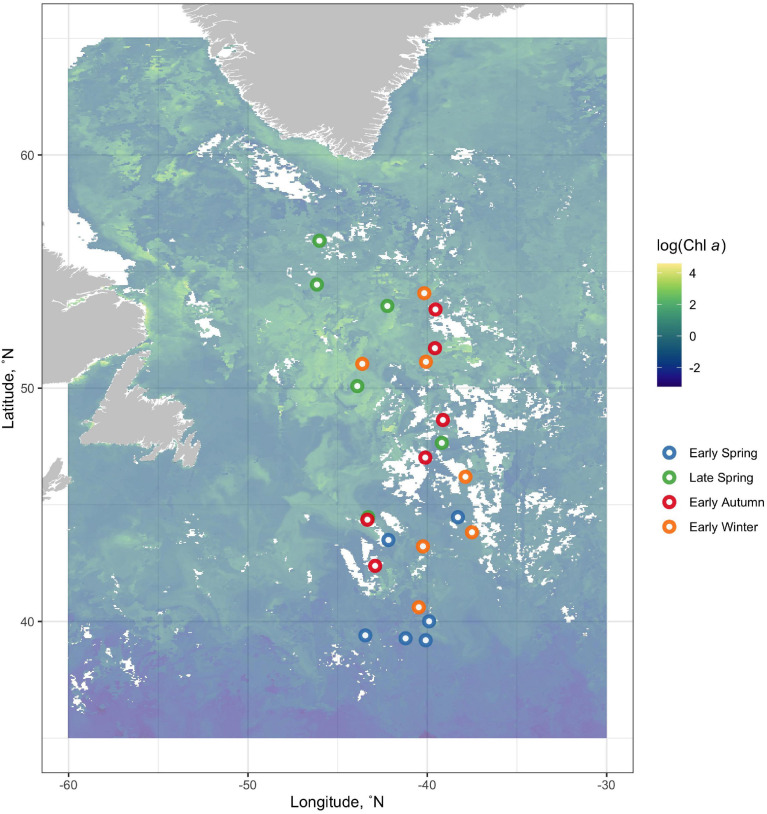
Station locations for all cruises are overlain on a map of 8-day composite log-transformed chlorophyll data from NASA’s Moderate Imaging Spectroradiometer (MODIS) collected during May 2016. NAAMES 1 occurred in the early winter (“winter transition”: November–December 2015, orange points), NAAMES 2 in the late spring (“climax transition”: May 2016, green points), NAAMES 3 in the early autumn (“depletion phase”: September 2017, red points), and NAAMES 4 in the early spring (“accumulation phase”: April 2018, blue points).

The NAAMES program, detailed in Behrenfeld et al. (2019), comprised of four field campaigns in the Western North Atlantic involving ship transects between 39 and 56°N latitude and −38 to −47°W longitude all aboard the R/V *Atlantis*. NAAMES was designed to resolve the dynamics and drivers of the annual phytoplankton bloom and their subsequent impacts on the atmosphere. Each cruise accordingly took place at a different time of the year at four different phases of the annual phytoplankton bloom cycle. NAAMES 1 occurred in the early winter (“winter transition”: November–December 2015), NAAMES 2 in the late spring (“climax transition”: May 2016), NAAMES 3 in the early autumn (“depletion phase”: September 2017), and NAAMES 4 in the early spring (“accumulation phase”: April 2018). Station locations for all cruises are overlaid on a map of 8-day composite chlorophyll data from NASA’s Moderate Imaging Spectroradiometer (MODIS) collected during May 2016 (Figure 1). These data (ID: erdMH1chla8day) were retrieved from NOAA’s ERDDAP servers^1^ using the package *rerddap* (v 0.7.4) in R (v.0.4.0).

### *In situ* Environmental Data

All processed data, analyses, and code presented here are available on GitHub^2^. NAAMES field cruise data are available through NASA’s SeaWiFS Bio-optical Archive and Storage System (SeaBASS)^3^ and the Biological & Chemical Oceanography Data Management Office (BCO-DMO, DOI: 10.26008/1912/bco-dmo.824623.1). All seawater samples were collected on the R/V *Atlantis* from 24 10-L Niskin bottles affixed to a Sea-Bird Scientific SBE-911+ Conductivity-Temperature-Depth rosette.

### Bacterioplankton Abundance

Bacterioplankton abundance (BA; cells l^–1^) were determined over four to eight depths throughout the euphotic zone, which ranged from 52 to 236 m. Cells were enumerated via flow cytometry on NAAMES 1 and via epifluorescence microscopy on the remaining cruises. Whole seawater was collected into sterile conical centrifuge tubes. Flow cytometry samples (2 ml) were preserved with 40 μl of 8% paraformaldehyde (Electron Microscopy Sciences) added to each sample to a final concentration of 0.2%. Samples were then mixed by inversion, flash frozen with liquid nitrogen, and stored at −80°C until analysis (Halewood et al., 2012). Microscopy samples were preserved with certified ACS formalin to a final concentration of 1% (vol:vol) and stored at 4°C until slide preparation within 36 h of collection. These preserved samples were filtered under gentle vacuum (∼34 kPa) onto 25-mm 0.2-μm polycarbonate (PC) membrane filters stained with Acid Black 107 (Irgalan Black) (Hobbie et al., 1977). Cells were stained with 4’,6-diamidino-2-phenylindole dihydrochloride (5 mg ml^–1^, DAPI) under minimal lighting according to Porter and Feig (1980). Filters were mounted onto slides with high-viscosity immersion oil (Thermo Scientific Richard-Allan Scientific Resolve) and stored at –20°C until enumeration at sea and at a shore-based laboratory. An Olympus BX51 epifluorescence microscope with ultraviolet excitation at 1,000× magnification was used to enumerate bacterioplankton cell abundances following Parsons et al. (2012). Briefly, 12 fields of view were counted for each slide and, on average, 50–60 cells were counted for each field of view.

Flow cytometry was performed following Halewood et al. (2012) using an LSR II equipped with a 488-nm blue laser and a high-throughput sampler [Becton Dickinson (BD) Biosciences]. Upon analysis, the LSR II was prepared according to the manufacturer’s guidelines. Spherotech Rainbow calibration beads (RCP-30-5) were used according to the manufacturer’s recommendations to diagnose cytometer laser detection performance. Samples were processed in batches of 45, which were thawed, vortexed, transferred to a 96-well plate, and stained with SYBR Green (100× dilution of commercial stock, Molecular Probes, Inc.) to a final concentration of 1:10,000 (vol:vol). To ensure complete staining of bacterioplankton cells, the plate was incubated for 15–30 min in darkness prior to analysis on the LSR II. Each well was analyzed for up to 90 s, with the minimum green FITC (fluorescein isothiocyanate) threshold set to 200 nm. The population of bacterioplankton cells (events) on the flow cytograms was interactively defined with a gate based on the relationship between side scatter and FITC fluorescence using FACSDiva software (BD Biosciences). Bacterioplankton abundance was calculated from the volume analyzed and the number of events in the gate. Internal references, consisting of a five-point serial dilution (dilution factor of 0.5) of surface Santa Barbara Channel seawater, were used to diagnose machine performance prior to staining NAAMES samples. This dilution series was prepared using whole seawater and 0.2 μm filtrate, and each dilution was enumerated via both flow cytometry and microscopy at the time of preparation. Several hundred 2-ml aliquots of the dilution series were fixed with 40 μl 8% paraformaldehyde and frozen at −80°C. Enumeration of these archived samples provided a means to assess daily machine performance such that large deviations between cell counts of original and archived samples indicated potential issues with machine fluidics or lasers. The slope (0.8) between the flow cytometry counts of these internal references at the time of sample analysis and the corresponding microscopy counts attained at the time of collection were used as a correction factor to align counts from the two enumeration methods. BA was integrated and normalized to the depth of the euphotic zone (i.e., 1% light level) for each station to obtain mean volumetric values.

### Net Bacterioplankton Production

Net BP rates were estimated by ^3^H-leucine (^3^H-Leu) incorporation using a modified version of the microcentrifuge method (Smith and Azam, 1992). For each depth, a killed control [killed immediately with 100 μl of 100% trichloroacetic acid (TCA)] and replicate 1.6-ml seawater samples were spiked with ^3^H-Leu (20 nM; specific activity 50.2–52.6 Ci/mmol; Perkin Elmer, Boston, MA, United States) and incubated for 2–3 h in the dark at ±2°C of *in situ* temperature. Incubations were terminated by adding 100 μl of cold 100% trichloroacetic acid (TCA) and were subsequently spun on a microcentrifuge at 20,800 × *g* for 7 min. The supernatant from each incubation tube was decanted, leaving a pellet that was then resuspended in 1.6 ml of 5% TCA. A second 7-min centrifugation step was performed, the supernatant again decanted, the remaining pellet resuspended in 1.6 ml of 80% ethanol (vol/vol), and finally centrifugation performed as described in Ducklow et al. (2001a). Ethanol was decanted and 1.6 ml of Ultima Gold scintillation cocktail added to each tube. Radioactivity was measured using a Hidex 300 Scintillation Analyzer and was corrected for quenching using an external gamma source and a quench curve. The coefficients of variation (CV) of assays performed following this protocol were generally 1–15% for replicate incubations; however, the deep samples generally had lower incorporation rates and CVs were often between 20 and 30%. ^3^H-Leu incorporation rates were converted to carbon units (μmol C l^–1^ d^–1^) using a conversion factor of 1.5 kg C (mol leucine incorporated)^–1^ (Simon and Azam, 1989). BP was integrated and normalized to the depth of the euphotic zone for each station to obtain mean volumetric values.

### Net Primary Production

Net Primary Production values (μmol C l^–1^ d^–1^) were determined using the photoacclimation productivity model (PPM) as reported in Fox et al. (2020). The PPM is based on quantitative understanding of shifts in phytoplankton chlorophyll synthesis in response to available light and nutrients (Behrenfeld et al., 2016). These photoacclimation responses were used to estimate depth-resolved phytoplankton growth rates and NPP at each NAAMES station. The PPM results exhibited strong agreement with 24-h ^14^C-uptake measurements of NPP determined at all stations occupied during the NAAMES campaign (Fox et al., 2020). NPP was integrated and normalized to the depth of the euphotic zone for each station to obtain mean volumetric rate values.

### Dissolved Organic Carbon Remineralization Experiments

**FIGURE 2:**
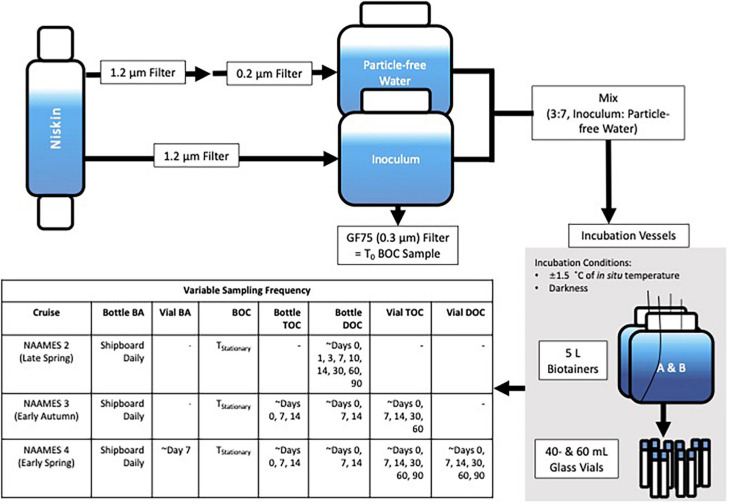
Schematic of the initiation and incubation conditions of the DOC remineralization experiments conducted on NAAMES 2–4, detailed in section “Dissolved organic carbon Remineralization Experiments.” Also included is a table of the sampling frequency for the incubations, in which bacterioplankton abundance is denoted by “BA,” bacterioplankton organic carbon by “BOC,” total organic carbon by “TOC,” and dissolved organic carbon by “DOC.” “*T*_0_” refers to the initial condition of the experiment while “*T*_*Stationary*_” refers to the stationary phase of cell growth determined by the bacterioplankton abundance curve. Note that *T*_0_ BOC was collected from the inoculum while *T*_*Stationary*_ BOC was collected from the bottle incubations.

At each station, experiments were conducted to determine the magnitude and rate of DOC remineralization using water collected from within the surface 10 m (see Figure 2 for experimental design and sampling scheme). Data from DOC remineralization experiments are available from BCO-DMO (doi: 10.26008/1912/bco-dmo.824623.1). Water was gently gravity-filtered through 142-mm PC filtration cartridges (Geotech Environmental Equipment, Inc.) loaded with either a 1.2- or a 0.2-μm mixed cellulose ester membrane filter (EMD Millipore). The 1.2-μm filtrate was retained as a bacterioplankton inoculum, and the 0.2-μm fraction was retained as particle-free media. The 1.2-μm filtrate retained on average 78 ± 16% of the bacterioplankton population in whole seawater (Supplementary Table 1). When possible, the filter cartridge was attached directly to Niskin bottles with platinum-cured silicone tubing, and the filtrate was collected into PC carboys. Otherwise, unfiltered water from the Niskin was first drawn into an acid-washed (10% HCl) and sample-rinsed PC carboy and then filtered into another acid-washed and sample-rinsed PC carboy. Each experiment was initiated by combining the 1.2-μm filtrate (inoculum) with the 0.2-μm filtrate at a 3:7 ratio. A pair of acid-washed PC incubation bottles (modified 5-l Nalgene Biotainer, Supplementary Material) was then rinsed with this water and subsequently filled. All carboys, tubing, and filtration rigs were rinsed with 10% HCl, then with Nanopure water, and finally with sample water three times before use. Membrane filters were flushed with a minimum of 1 l of Nanopure water followed by 0.5 l of sample water prior to collecting filtrate to minimize DOC leaching from the filters.

The experiments performed incubation in the dark and within ±1.5°C of *in situ* temperatures using refrigerated incubators (Fisherbrand Isotemp BOD). On NAAMES cruises 3 and 4, precombusted (4 h at 450°C) 40- and 60-ml borosilicate glass incubation vials (Thermo Scientific) were also rinsed and filled with initial incubation water and served as parallel incubations to monitor changes in organic carbon. After returning to port, the vials were shipped overnight in coolers to UC Santa Barbara, transferred to incubators at *in situ* temperatures, and periodically sampled for up to 110 days (*T*_*End*_) after the initiation of the experiment.

Throughout the duration of each experiment, samples for BA, bacterioplankton organic carbon (BOC), total organic carbon (TOC), and DOC, were collected using the positive pressure displacement system (Supplementary Material; Liu et al., 2020). While at sea, Bacterioplankton abundance samples were monitored daily, BOC samples were collected at the initiation time for each experiment (*T*_0_) and during the stationary phase (*T*_*Stationary*_) of the bacterioplankton growth curve, and TOC and DOC samples were collected three to six times within the first 14 days. DOC samples were also collected after returning to port from NAAMES 2 and 4 at the 1-, 2-, and 3-month marks. TOC samples were collected at the 1- and 2-month marks after returning to port from NAAMES 3 and after the 1-, 2-, and 3-month marks after NAAMES 4. Bacterioplankton abundance and BOC were not collected after returning to port from the different cruises.

For each experiment, BOC samples were collected from the 1.2-μm filtrate (inoculum) at *T*_0_ and from each incubation bottle at *T*_*S*__*tationary*_ of the bacterioplankton growth curve. *T*_0_ BOC samples were collected from the 1.2-μm filtrate instead of from the mixed experimental water (3:7 inoculum: particle free water) to ensure that enough cellular material was collected for later CHN analysis, described below. Thus, T_0_ BOC in the incubation bottles was estimated as 30% of the BOC measured from the 1.2-μm filtrate. For each BOC sample, 1 l of seawater was drawn from a Biotainer and filtered through a polypropylene inline filter cartridge (Cole-Parmer) loaded with two combusted Advantec Grade 25-mm 0.3-μm glass fiber filters (GF75) (Stephens et al., 2020). Two filters were used to increase cell retention (mean 78 ± 9%). Each filter was folded twice, with the sample material on the inside, placed into separate precombusted (450°C for 4 h) 20-ml borosilicate glass vials (Wheaton), and frozen at −20°C. Filters were analyzed on a Costech ECS 4010 CHNS-O elemental analyzer by Bigelow Analytical Services, which has a detection limit of 0.1 μg C (Bigelow Laboratory for Ocean Sciences, Maine). At each station of NAAMES 2 and 4, 1 l of either 0.2-μm or 30-kDa tangential flow-filtration (TFF, EMD Millipore) 10-m seawater filtrate was also filtered through a pair of stacked GF75s. The TFF filtrate did not contain any particles greater than 30 kDa; thus, any organic matter retained on the GF75 filter after passing 1 l of TFF filtrate through it was considered absorbed DOC. There was no significant difference between absorbed DOC estimates from filters treated with 0.2 μm or 30 kDa TFF seawater (Wilcoxon *p* = 0.1). The average absorbed DOC estimate, 2.7 μg, was used as a universal blank. For reference, the average carbon value on the second (i.e., bottom) GF75 filter of the experimental samples was 5.1 ± 3.5 μg, indicating the utility of using two GF75 filters to increase the retention of cell carbon.

Both a filtered DOC sample and a TOC sample were collected to monitor changes in organic carbon over the course of each experiment. TOC samples were corrected by the contribution of BOC at *T*_0_ and *T*_*Stationary*_, hereafter referred to as DOC^∗^ (Stephens et al., 2020; Wear et al., 2020). BA was observed to fall to low densities by *T*_*E*__*nd*_ (Supplementary Figure 1), so we considered TOC and DOC to be interchangeable by *T*_*E*__*nd*_. In the absence of BOC collections after *T*_*Stationary*_, we did not calculate DOC^∗^ at *T*_*E*__*nd*_ because doing so may have artificially enhanced estimates of DOC removal. On NAAMES 2, filtered DOC samples were displaced by positive pressure from each 5-l Biotainer through an inline set of two precombusted GF75 filters and into two pre-combusted 40-ml borosilicate glass vials. The collected volume was then immediately fixed by adding 50 μl of DOC-free 4-N HCl to a pH of ∼3. We not only observed relatively clean sampling of DOC filtered directly from the Biotainers during NAAMES but also found greater variability in the temporal trends of DOC concentration when the incubation water volume was less than half of the original incubation volume, after large volumes were removed for DNA and BOC samples at *T*_*Stationary*_ (Supplementary Figure 2). In order to address this issue and reduce potential contamination by handling on subsequent cruises (NAAMES 3 and 4), parallel 40- and 60-ml borosilicate glass incubation vials were added to monitor changes in bulk TOC and DOC. The adoption of these vials not only helped address potential contamination from handling but also permitted the long-term monitoring of DOC removal as vials were sampled periodically until *T*_*End*_ (Stephens et al., 2020). A direct comparison conducted on both NAAMES 3 and 4 indicated that the filtered DOC concentrations and corresponding DOC^∗^ estimates were within 10% of one another, with a systematic positive bias of the filtered DOC measurements relative to DOC^∗^ (Supplementary Figure 3). On NAAMES 4, bacterioplankton abundance and DOC estimates from the large-volume Biotainer and the parallel vials at corresponding timepoints were within 7 and 5% of one another, respectively, indicating that both incubation containers tracked similar microbial dynamics (Supplementary Figure 4). Thus, all analyses described below used filtered DOC for NAAMES 2 experiments and DOC^∗^ for NAAMES 3 and 4 experiments.

Organic carbon concentrations were determined via the high-temperature combustion method using modified Shimadzu TOC-V or TOC-L analyzers as described in Carlson et al. (2010). Concentrations were quantified using standard solutions of glucose and ultrapure (low-carbon) water. All samples were systematically referenced against surface (5-m) water and deep (<2000-m) Atlantic seawater that were calibrated against consensus reference material (Hansell SSR Lot#08-18) and run every six to eight samples (Hansell and Carlson, 1998). Typical run sizes were kept under 35 samples to reduce salt accumulation and instrument drift. The precision of the Shimadzu analyzers for surface samples was within 2% CV.

### Calculations of Derived Variables

#### Seasonally Accumulated DOC Bioavailability and Persistence

The microbial dynamics and DOC bioavailability detailed in this manuscript are placed in the context of seasonally accumulated DOM for each station and cruise. The magnitude of DOC that accumulated in excess of the annual DOC minimum that corresponded to the maximal deep winter mixing was determined for each station and is referred to as seasonally accumulated DOC (DOC_*SA*_). The annual DOC minimum was approximated for each 1° latitudinal bin of the NAAMES study region according to and as reported by Baetge et al. (2020). Briefly, observed profiles of DOC concentration at each station were redistributed over their corresponding local maximum MLD that were retrieved from ARGO float observations between May 5, 2014, and December 2, 2018 (Table 1). For each DOC remineralization bioassay, DOC_*SA*_, μmol C l^–1^ was then calculated as the difference between the initial DOC concentration and the annual DOC minimum. Bioavailable DOC (ΔDOC) for each DOC remineralization experiment was calculated as the total removal of DOC_*SA*_ over the short term (*T*_0_ – *T*_*Stationary*_) and long term (*T*_0_ – *T*_*End*_), expressed as both concentration (μmol C l^–1^) and percentage of initial DOC_*SA*_. ΔDOC removal rates (μmol C l^–1^ d^–1^) for the DOC remineralization experiments were calculated as ΔDOC divided by the number of elapsed days. DOC_*SA*_ that persists was calculated as concentration (μmol C l^–1^) and percent of DOC_*SA*_ remaining at *T*_*End*_. It is important to note that DOC_*SA*_ may be comprised of DOC compounds that had accumulated in previous seasons. Thus, DOC removal observed in the early autumn experiments may have reflected the removal of DOC that had accumulated earlier in the spring, not necessarily solely DOC produced in the autumn.

**TABLE 1:** BGEs and DOC bioavailability from DOC remineralization experiments.

Station	Latitudinal bin (°N)	Longitudinal bin (°W)	Max MLD (m)	Annual DOC minimum (μmol C l^–1^)	*T*_0_ DOC (μmol C l^–1^)	*T*_0_ DOC_*SA*_ (μmol C l^–1^)	*T*_*Stationary*_ (days)	*T*_*end*_ (days)	*T*_0_ BOC (μmol C l^–1^)	*T*_*Stationary*_ BOC (μmol C l^–1^)	ΔBOC (μmol C l^–1^)	*T*_*Stationary*_ DOC (μmol C l^–1^)	Short-term ΔDOC (μmol C l^–1^)	BGE	Short-term %ΔDOC (μmol C l^–1^)	Short-term ΔDOC removal rate (μmol C l^–1^ d^–1^)	*T*_*End*_ DOC_*SA*_ (μmol C l^–1^)	Long-term ΔDOC (μmol C l^–1^)	Long-term %ΔDOC (μmol C l^–1^)	Long-term ΔDOC removal rate (μmol C l^–1^ d^–1^)	Persistent DOC_*SA*_ (μmol C l^–1^)	% Persistent DOC_*SA*_
***Early Spring (April 2018): “Accumulation Phase”***																						
1	39	−44	294	55.5	60.3 ± 0.6	4.8	8	84	0.1	1.1 ± 0.0	1.0 ± 0.0	56.9 ± 0.9	3.4 ± 0.2	0.30 ± 0.01	71 ± 5	0.4 ± 0.0	55.6 ± 0.2	4.7 ± 0.8	99 ± 18	0.06 ± 0.01	0	0
2	39	−41	294	55.5	58.3 ± 0.1	2.8	6	83	0.2	0.8 ± 0.1	0.6 ± 0.1	57.6 ± 0.3	NR	NR	NR	NR	55.4 ± 0.1	3.1 ± 0.0	111 ± 0	0.04 ± 0.0	0	0
2RD	39	−40	294	55.5	59.5 ± 0.1	4.0	7	97	0.2	1.7 ± 0.1	1.5 ± 0.4	57.0 ± 0.5	NR	NR	NR	NR	55.8 ± 0.6	3.8 ± 0.8	95 ± 20	0.04 ± 0.01	0	0
2RF	39	−40	294	55.5	58.5 ± 0.0	2.4	7	96	0.1	1.2 ± 0.0	1.1 ± 0.0	56.7 ± 0.3	NR	NR	NR	NR	54.5 ± 0.1	4.0	167	0.04	0	0
3	44	−42	368	55.9	58.2 ± 0.3	2.3	9	82	0.1	0.7 ± 0.1	0.6 ± 0.1	57.7 ± 0.5	NR	NR	NR	NR	55.3 ± 0.3	3.1 ± 0.6	132 ± 28	0.04 ± 0.01	0	0
4	44	−38	368	55.9	56.7 ± 0.3	0.8	9	100	0.1	0.6 ± 0.1	0.5 ± 0.1	53.9 ± 0.6	2.8	0.21	350	0.3 ± 0.1	54.1 ± 0.7	2.3	288	0.02	0	0
Overall (mean, SD)			319 ± 38	55.6 ± 0.2	58.6 ± 1.2	2.9 ± 1.4	–	–	0.1 ± 0.05	1.0 ± 0.4	0.9 ± 0.4	56.6 ± 1.4	3.2 ± 0.4	0.27 ± 0.05	211 ± 197	0.4 ± 0.1	55.1 ± 0.7	3.6 ± 0.9	149 ± 73	0.04 ± 0.01	0	0
***Late Spring (May 2016): “Climax Transition”***																						
5	44	−43	368	55.9	65.7 ± 0.5	9.8	6	104	0.5	1.1 ± 0.1	0.6 ± 0.1	63.1 ± 0.8	2.6 ± 0.3	0.21	27 ± 7	0.4 ± 0.1	61.7 ± 1.1	4.0 ± 1.1	41 ± 11	0.04 ± 0.01	5.8 ± 1.1	59 ± 11
4	48	−39	508	51.3	54.7 ± 1.1	3.4	7	8	0.2	0.7 ± 0.0	0.5 ± 0.0	52.6 ± 0.0	2.1	0.24	62	0.3	53.4 ± 0.8	NR	NR	NR	NR	NR
3	50	−44	406	52.4	60.3	7.9	10	13, 110	0.4	ND	ND	57.8 ± 0.6	2.5 ± 0.6	NR	32	0.3 ± 0.1	57.3 ± 0.6	3.0	38	0.03	4.9	62
2	54	−42	223	50.4	57.3 ± 0.4	6.9	8	14	0.5	1.5 ± 0.0	1.0 ± 0.0	56.5 ± 0.1	NR	NR	NR	NR	55.0 ± 0.1	2.6	38	0.2	4.3	62
1	56	−46	110	51.2	54.5 ± 1.0	3.3	8	17	0.4	1.0 ± 0.1	0.6 ± 0.1	51.9 ± 0.5	2.6 ± 0.5	0.22 ± 0.02	77 ± 15	0.3 ± 0.1	51.2 ± 0.6	3.3 ± 0.2	98 ± 6	0.20 ± 0.01	0	0
Overall (mean, SD)			323 ± 157	52.0 ± 2.1	58.5 ± 4.7	6.3 ± 2.9	–	–	0.4 ± 0.1	1.1 ± 0.3	0.6 ± 0.2	56.3 ± 4.6	2.5 ± 0.4	0.22 ± 0.01	50 ± 24	0.3 ± 0.1	55.7 ± 4.0	3.3 ± 0.7	59 ± 31	0.1 ± 0.09	3.8 ± 2.6	46 ± 31
***Early autumn (September 2017): “Depletion Phase”***																						
1	42	−43	418	55.4	66.8 ± 0.2	11.4	7	69	0.2	2.1 ± 0.4	1.9 ± 0.4	62.5 ± 0.1	4.3 ± 0.1	0.45 ± 0.11	37 ± 1	0.6 ± 0.0	62.2 ± 0.7	4.8 ± 1.2	42 ± 11	0.07 ± 0.02	6.6 ± 1.2	58 ± 11
2	44	−43	368	55.9	74.3 ± 0.7	18.4	7	68	0.5	2.5 ± 0.1	2.0 ± 0.0	73.7 ± 0.6	NR	NR	NR	NR	68.9 ± 1.2	5.8 ± 0.8	32 ± 5	0.09 ± 0.01	12.6 ± 0.8	68 ± 5
3	47	−40	336	54.2	68.8 ± 0.8	14.6	5	66	0.7	1.6 ± 0.4	0.9 ± 0.4	66.1 ± 1.9	2.7	0.22	18	0.5	64.0 ± 0.2	4.5 ± 1.3	31 ± 10	0.07 ± 0.02	101 ± 1.3	69 ± 10
4	49	−39	448	51.6	66.1 ± 0.4	14.5	7	64	0.6	1.0 ± 0.1	0.4 ± 0.1	64.8 ± 0.4	NR	NR	NR	NR	62.9 ± 0.2	3.8 ± 0.7	26 ± 5	0.06 ± 0.01	10.7 ± 0.7	73 ± 5
5	52	−40	330	56.0	68.8 ± 0.6	12.8	7	62	1.4	1.4 ± 0.2	0.1 ± 0.2	68.0 ± 0.2	NR	NR	NR	NR	65.0 ± 0.4	5.3 ± 1.1	41 ± 8	0.08 ± 0.02	7.6 ± 1.1	59 ± 8
6	53	−40	231	52.7	60.5 ± 0.3	7.8	8	59	1.1	1.6 ± 0.3	0.5 ± 0.3	57.9 ± 0.1	2.6 ± 0.2	0.20 ± 0.09	33 ± 3	0.3 ± 0.0	56.6 ± 1.0	5.0 ± 1.1	64 ± 15	0.08 ± 0.02	2.8 ± 1.1	35 ± 15
Overall (mean, SD)			355 ± 76	54.2 ± 1.8	67.9 ± 4.5	13.3 ± 3.6	–	–	0.8 ± 0.4	1.7 ± 0.5	1.0 ± 0.8	65.5 ± 5.3	3.2 ± 0.9	0.30 ± 0.15	29 ± 10	0.4 ± 0.1	63.2 ± 4.0	4.8 ± 1.0	40 ± 15	0.08 ± 0.02	8.4 ± 3.5	60 ± 14

Station: where experiments were conducted; Latitudinal bin: stations were binned to the nearest degree based on latitudinal coordinates; Longitudinal bin: binned to the nearest degree; Max MLD: maximum mixed layer depth identified from ARGO profiling data between 2014 and 2018 for each latitudinal bin, detailed in Baetge et al. (2020); Annual DOC minimum: DOC concentration corresponding to the maximal deep winter mixing, which was determined for each station as detailed in Baetge et al. (2020); DOC_*SA*_: Seasonally accumulated DOC, magnitude of DOC that accumulated in excess of annual DOC minimum; *T*_0_ DOC: initial DOC concentration in the DOC remineralization experiments; DOC_*SA*_: seasonally accumulated DOC, calculated as *T*_0_ DOC minus the corresponding annual DOC minimum; *T*_*Stationary*_: stationary growth phase timepoint where BOC and DOC(*) samples were collected; *T*_*End*_: termination of experiments; *T*_0_ BOC: BOC at *T*_0_; *T*_*Stationary*_ BOC: BOC at *T*_*Stationary*_; ΔBOC: change in BOC from *T*_0_ to *T*_*Stationary*_; *T*_*Stationary*_ DOC: DOC at *T*_*Stationary*_; Short-term ΔDOC: change in DOC from *T*_0_ to *T*_*Stationary*_ equal to bioavailable DOC, expressed as concentration, %DOC_*SA*_, and removal rate; *T*_*End*_ DOC: DOC at *T*_*End*_; Long-term ΔDOC: change in DOC from *T*_0_ to *T*_*End*_ equal to bioavailable DOC, expressed as concentration, %DOC_*SA*_, and removal rate; Persistent DOC_*SA*_: the fraction of DOC_*SA*_ remaining at *T*_*End*_, that is, DOC_*SA*_; Long-term ΔDOC, expressed as concentration and %DOC_*SA*_. Data represent means of replicated experiments with error representing standard deviations. NR = Not Resolvable due to DOC or TOC removal not meeting criteria of ≥ 2 μmol C l^–1^; ND = No Data.

#### Bacterioplankton Growth Efficiencies

Bacterioplankton growth efficiency (BGE) was determined by assessing changes in BOC from *T*_0_ to *T*_*Stationary*_ relative to the drawdown of DOC over the same time frame in each DOC remineralization experiment. BGE was determined by the following equation:

(1) $$\text{BGE}=\frac{{\text{BOC}}_{\text{Stationary}}-{\text{BOC}}_{\text{Initial}}}{{\text{DOC}}_{\text{Initial}}-{\text{DOC}}_{\text{Stationary}}}$$

where the difference in BOC between *T*_0_ and *T*_*Stationary*_ (ΔBOC) was calculated as the change in the total carbon concentration captured on the GF75 filters divided by the simultaneous change in DOC or DOC^∗^ (ΔDOC). The change in BOC (ΔBOC) was not calculated for experiments conducted at Station 3 in the late spring because of a lack of a BOC sample at *T*_*Stationary*_. BGEs are highly sensitive to changes in DOC and can be artificially inflated when ΔDOC is small; thus, we chose to be conservative by only calculating BGEs for experiments where the removal of DOC was greater or equal to 2 μmol C l^–1^.

#### Bacterioplankton Carbon Demand

For each cruise, BCD (μmol C l^–1^ d^–1^) was calculated as integrated, depth-normalized *in situ* BP divided by the campaign-wide mean BGE (0.26) calculated from all cruises. The fraction of *in situ* NPP that can potentially support *in situ* BCD is expressed as BCD:NPP.

### Statistics

All statistical analyses were performed using packages in R (v 4.0.0). Bland–Altman/Tukey mean-difference analyses were used to assess the agreement between corresponding experimental measurements of DOC and DOC^∗^ as well as the dynamics between experimental incubation containers. Agreement statistics were computed using the function *blandr.statistics* from the package *blandr* (v 0.5.1). The correlation between these measurements was also evaluated using standardized (reduced) major axis model II linear regressions. Standardized (reduced) major axis model II linear regressions were also used to explore the relationship between BCD and NPP. Regressions were computed using the function *lmodel2* from the package *lmodel2* (v 1.7-3). Model fits with *p*-values >0.05, ≤0.05, or ≤0.01 are described as “not significant,” “significant,” and “highly significant,” respectively.

A non-parametric Kruskal–Wallis test (one-way ANOVA on ranks) was performed on each bacterioplankton growth metric, as well as on NPP, to assess if means across all seasons were equivalent. If they were not equivalent, a nonparametric Wilcoxon test was then performed *post hoc* to assess whether means between two seasons were equal. Kruskal–Wallis and Wilcoxon tests were both performed using the *compare_means* function in the package *ggpubr* (v 0.3.0). For both tests, group means that were likely equal, significantly different, or highly significantly different are indicated by *p*-values >0.05, ≤0.05–0.01, and ≤0.01, respectively.

## Results

### Bacterioplankton and DOM Dynamics in the Remineralization Experiments

**FIGURE 3:**
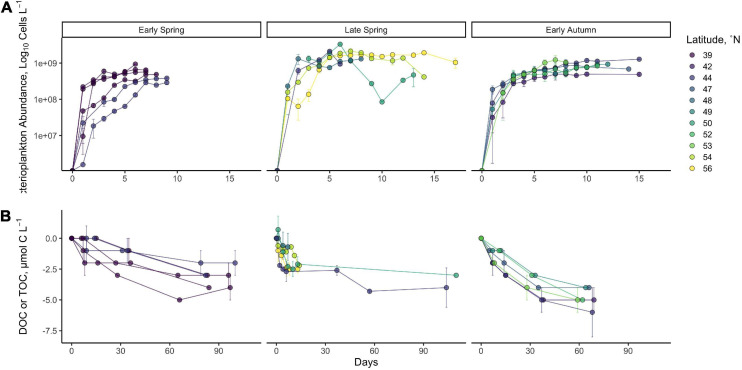
Time series of station mean log_10_ cell abundance **(A)** and DOC/TOC **(B)** from DOC remineralization experiments. Data represent the change in bacterioplankton log_10_ cell abundance from *T*_0_ up to 17 days or change in DOC concentration throughout the long-term incubation of up to 110 days. Late-spring organic carbon data are from filtered DOC samples, while early spring and early autumn organic carbon data are from unfiltered TOC samples. Error bars indicate the standard deviation between the means of replicated experiments.

Bacterioplankton generally followed the logistic model of growth (Figure 3A). While bacterioplankton cell production was highly variable within each season, the general trend indicated a greater change in cell abundance in late spring relative to the other seasons. It is notable, however, that the change in BOC measured from *T*_0_ to *T*_*Stationary*_ was not statistically different between seasons (Table 1, *p* = 0.44). This may result from differences in cell size (not measured) or carbon per cell between different seasons.

Dissolved organic carbon remineralization was also variable within each season and between seasons (Figure 3B). Total short-term DOC removal, between *T*_0_ and *T*_*Stationary*_ (5–10 days of incubation) in each experiment, was limited to a range of 2.1–4.3 μmol C l^–1^ across all seasons (Figure 3B), with no statistical difference in the mean magnitude of DOC removed between seasons (Figure 3B, Table 1, Kruskal–Wallis *p* = 0.13).

### The Bioavailable Fraction of Seasonally Accumulated DOC From DOC Remineralization Experiments

On average, the magnitude of DOC above annual surface minimum DOC concentration (i.e., DOC_*SA*_) increased as stratification of the water column intensified from the early spring (2.9 ± 1.4 μmol C l^–1^), to late spring (6.3 ± 2.9 μmol C l^–1^), and to early autumn (13.3 ± 3.6 μmol C l^–1^) (Table 1). Here, we used the DOC removal from remineralization experiments to assess the bioavailable fraction of this surface accumulated pool (ΔDOC, Figure 3B). Over the spatial extent of the NAAMES study region and the temporal period between the early spring and early winter, the magnitude of short-term ΔDOC (i.e., short-term DOC removal within 10 days) lacked any clear seasonal trends, as noted above. Latitudinal trends in short-term ΔDOC were also absent within individual seasons (Kruskal–Wallis *p* = 0.22 [early spring], *p* = 0.68 [late spring], *p* = 0.19 [early autumn]). Long-term ΔDOC (*T*_0_–*T*_*End*_, 13–110 days) for the spring averaged 3.4 ± 0.9 and 4.8 ± 1.0 μmol C l^–1^ for early autumn (Table 1). The long-term ΔDOC was not significantly different from spring to autumn; however, there were large seasonal differences in proportion of the ΔDOC relative to the amount of accumulated DOC (ΔDOC:DOC_*SA*_). ΔDOC in the short term was greatest in the early spring, representing on average 211 ± 197% of the DOC_*SA*_ pool, before decreasing to 50 ± 24% in the late spring and then to 29 ± 10% in the early autumn. Over the long term, a similar trend was observed in the incubations whereby the percentage of DOC_*SA*_ that was bioavailable was greatest in early spring (>140%) and lowest (∼40%) in early autumn (Table 1). ΔDOC greater than 100% in our experiments indicated that the responding heterotrophic bacterioplankton community was not only able to degrade all of DOC_*SA*_ but also able to remove some fraction of the presumably lower-quality background DOC pool represented by the annual DOC minimum. It should also be noted that DOC contamination during post-cruise sampling in several of the late spring (NAAMES 2) experiments precluded accurate resolution of long-term DOC removal in four of the six sets of experiments for that cruise (Table 1). The source of DOC contamination in the long-term incubations remains unclear but may be related to the increase in container surface area to seawater volume ratio as incubation volume was drawn below 50% of initial volume (Supplementary Figure 2). Regardless, the trend of decreasing ΔDOC:DOC_*SA*_ from the early spring to the early autumn is evident from both the short-term and long-term estimates of DOC removal within the remineralization experiments.

**FIGURE 4:**
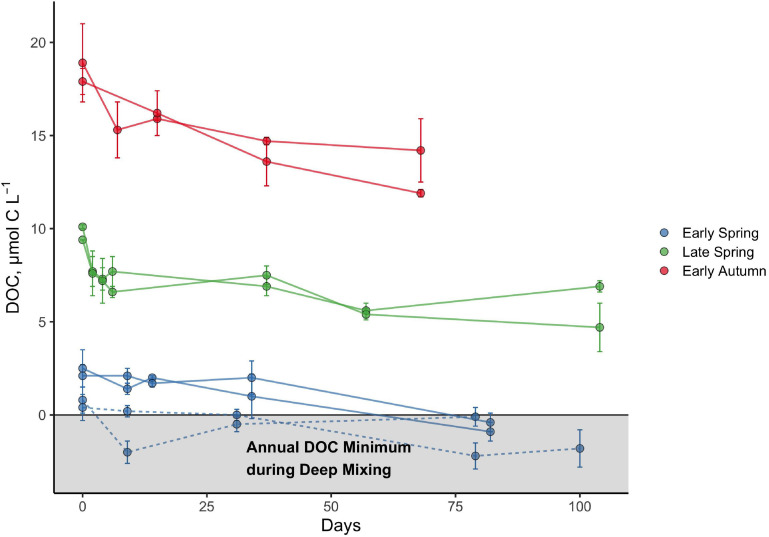
Bioavailability and persistence of seasonally accumulated DOC (DOC_*SA*_) at the 44°N latitudinal bin of the NAAMES study region based on DOC remineralization experiments. Experiments were conducted at two different stations corresponding to 44°N in the early spring and are denoted by the solid (station 3) and dashed lines (station 4). Data represent measurements for each incubation bottle, with error bars representing the standard deviations. The horizontal line intersecting the *y*-axis at 0 represents the baseline after the annual minimum DOC concentration corresponding to the maximal deep winter mixing (Baetge et al., 2020) has been subtracted from the measured surface DOC concentration for each season.

The seasonal progression of the relative percentage of DOC_*SA*_ that was bioavailable or that persisted was best exemplified at latitude 44°N, which was occupied for each of the early spring, late spring, and early autumn cruises (Figure 4). The initial condition of these experiments demonstrates the increasing magnitude of the total DOC_*SA*_ pool as well as the ΔDOC fraction from spring to early autumn. As described above, however, the relative contribution of ΔDOC was greatest in early spring and decreased by late spring and early autumn. Conversely, the fraction of the DOC_*SA*_ pool persisting after ∼60 days increased from spring to early autumn. In many cases, all of the DOC_*SA*_ determined in the spring was bioavailable on the time scales of the remineralization experiment, whereas up to 73% of the DOC_*SA*_ pool determined for early autumn persisted over the experimental incubation period (Table 1).

### Bacterioplankton Growth Efficiencies From DOC Remineralization Experiments

**FIGURE 5:**
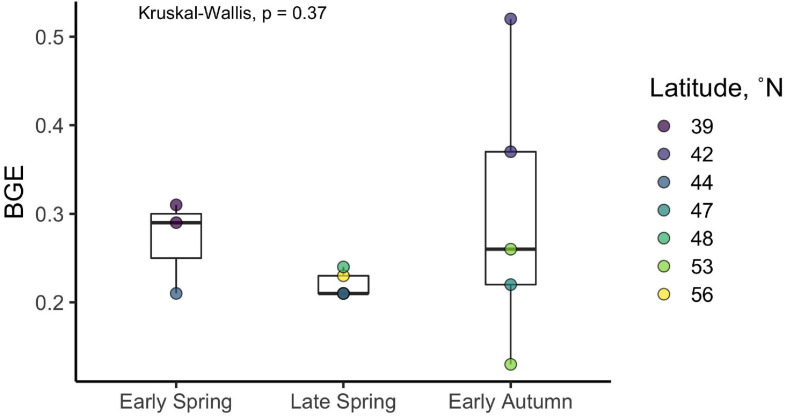
Bacterioplankton growth efficiency (BGE) estimates derived from the DOC remineralization experiments. Filled circles represent individual incubations, and filled colors represent the station where the experiment was conducted (see Table 1 for corresponding station latitudes). Boxes represent the 1.5 interquartile range, with the internal solid line representing the median. *p*-values are reported for the nonparametric Kruskal–Wallis test (one-way ANOVA on ranks), which tests if the means of all groups are equal.

The ratio of the change in BOC from *T*_0_ to *T*_*Stationary*_ to the corresponding change in DOC over the time frame of the short-term incubations was used to derive estimates of BGE for each experiment (Figure 5 and Table 1). Although there was considerable variability in BGE within and between seasons, ranging from 0.13 to 0.52 with a mean of 0.26, no significant difference in BGE was found between seasons (Kruskal–Wallis *p* = 0.37) or latitudinal range (Kruskal–Wallis *p* = 0.15).

### *In situ* NPP and Bacterioplankton Growth Metrics

**FIGURE 6:**
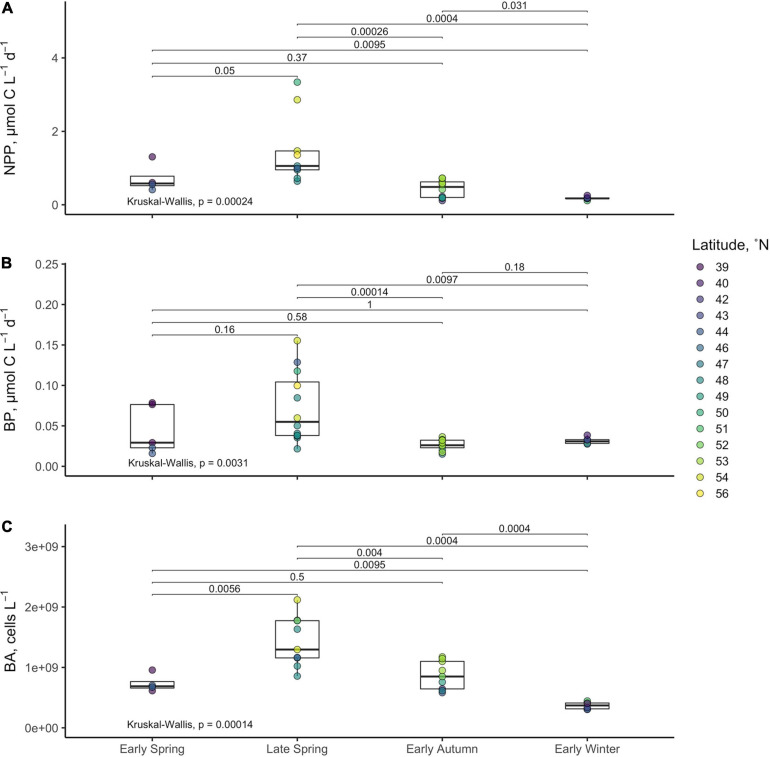
Averages of net primary production [NPP **(A)**], bacterioplankton production [BP **(B)**], and bacterioplankton abundance **(C)** within the euphotic zone for each station. Boxes represent the 1.5 interquartile range, with the internal solid line representing the median. Circles represent data points. *p*-values are reported for the nonparametric Kruskal–Wallis test (one-way ANOVA on ranks), which tests if the means of all groups are equal. Level of significance is also reported for the nonparametric two-sample Wilcoxon test, which tests whether the means between two groups are equal.

Average NPP rates estimated over the euphotic zone, ranged from 0.11 to 3.34 μmol C l^–1^ d^–1^ and declined significantly from late spring to early winter (Figure 6 and Table 2). Metrics of bacterioplankton abundance and production over the euphotic zone demonstrated similar seasonal trends, such that the range of variability and the magnitude of rates or cell densities were greatest in late spring and lowest in autumn/winter. Significant temporal differences in each of the bacterioplankton growth metrics were primarily driven by the decline of those parameters from the spring to the early autumn. Bacterioplankton abundance ranged from 3.04 × 10^8^ to 2.12 × 10^9^ cells l^–1^, increasing from the early to late spring before decreasing in early autumn (Figure 6). Net BP, ranging from 0.015 to 0.155 μmol C l^–1^ d^–1^, never exceeded NPP, and overall net BP represented 3–25% of NPP (Figure 6 and Table 2).

**TABLE 2:** NPP, BP, and BCD.

Station	Latitudinal Bin (°N)	MLD.(m)	Euphotic Zone Depth (m)	Temperature (°C)	Chl *a* (mg m^–3^)	NPP (μmol C l^–1^d^–1^)	BP (μmol C l^–1^d^–1^)	BP:NPP (%)	BCD (μmol C l^–1^d^–1^)	BCD:NPP (%)
***Early Spring (April 2018): “Accumulation Phase”***										
1	39	80	106	18.3	0.67	0.60	0.03	5	0.11	19
2	39	59 ± 7	98	17.0 ± 0.01	0.73 ± 0.26	1.31	0.08 ± 0.001	6	0.30 ± 0.006	23
3	44	214	120	18.2	0.64	0.41	0.02	6	0.09	21
4	44	129	126	13.1	0.57	0.56	0.02	3	0.06	11
Overall (mean, SD)		121 ± 69	113 ± 13	16.7 ± 2.1	0.67 ± 0.15	0.84 ± 0.43	0.05 ± 0.03	5 ± 1	0.17 ± 0.12	19 ± 5
***Late Spring (May 2016): “Climax Transition”***										
5	44	34	91	15.8	1.07	0.99	0.13	13	0.50	50
4	48	73 ± 104	116 ± 22	15.5 ± 0.03	0.85 ± 0.30	0.82 ± 0.16	0.04 ± 0.02	5 ± 1	0.17 ± 0.08	20 ± 6
3	50	9	52	8.7	5.74	3.34	0.12	4	0.45	14
0	54	13	87	3.9	0.95	1.47	0.06	4	0.23	16
2	54	23	54	5.5	3.31	2.86	0.16	5	0.60	21
1	56	34	72	3.9	1.49	1.36	0.10	7	0.38	28
Overall (mean, SD)		31 ± 23	79 ± 24	12.2 ± 5.1	1.54 ± 1.52	1.32 ± 0.88	0.07 ± 0.04	6 ± 3	0.28 ± 0.17	22 ± 10
***Early Autumn (September 2017): “Depletion Phase”***										
1	42	38	244	18.0	0.18	0.11	0.03	23	0.10	87
2	44	38	207	8.0	0.21	0.23	0.03	14	0.12	55
3	47	17	200	16.6	0.13	0.18	0.02	8	0.06	32
4	49	40	188	10.4	0.17	0.19	0.02	11	0.08	41
5	52	17	157	8.7	0.39	0.42	0.03	6	0.10	24
6	53	35 ± 2	103 ± 6	7.3 ± 0.6	1.38 ± 0.37	0.66 ± 0.08	0.03 ± 0.01	4 ± 1	0.11 ± 0.03	17 ± 5
Overall (mean, SD)		30 ± 11	183 ± 48	9.6 ± 4.0	0.85 ± 0.67	0.46 ± 0.24	0.03 ± 0.01	8 ± 6	0.10 ± 0.03	31 ± 23
***Early Winter (November – December 2015): “Winter Transition”***										
7	40	27	98	18.3	0.57	0.25	0.04	15	0.15	58
6	43	7	103	17.1	0.49	0.19	0.03	17	0.13	67
5	44	79	103	16.2	0.48	0.17	0.03	20	0.13	75
4	46	101	126	15.5	0.45	0.17	0.03	16	0.11	62
3	51	64	59	7.2	1.41	0.11	0.03	25	0.11	96
2	54	95	104	5.5	0.87	0.18	0.03	16	0.11	61
Overall (mean, SD)		62 ± 38	98 ± 22	13.3 ± 5.5	0.71 ± 0.38	0.179 ± 0.045	0.03 ± 0.004	18 ± 4	0.12 ± 0.02	70 ± 14

*Station: where experiments were conducted; Latitudinal Bin: stations were binned to the nearest degree based on latitudinal coordinates; MLD: mixed layer depth; Euphotic Zone Depth: 1% light level; Temperature: CTD-recorded temperature integrated and normalized to the depth of the euphotic zone; Chl *a*: chlorophyll *a* estimated from CTD fluorescence, integrated and normalized to the depth of the euphotic zone; NPP: net primary production integrated and normalized to the depth of the euphotic zone; BP: net bacterioplankton production integrated and normalized to the depth of the euphotic zone; BP:NPP: the ratio of BP to NPP; BCD: bacterioplankton carbon demand or gross bacterioplankton production integrated and normalized to the depth of the euphotic zone. BCD:NPP: the ratio of BCD to NPP. Data represent means of estimates from multiple profiles with error representing standard deviations.*

**FIGURE 7:**
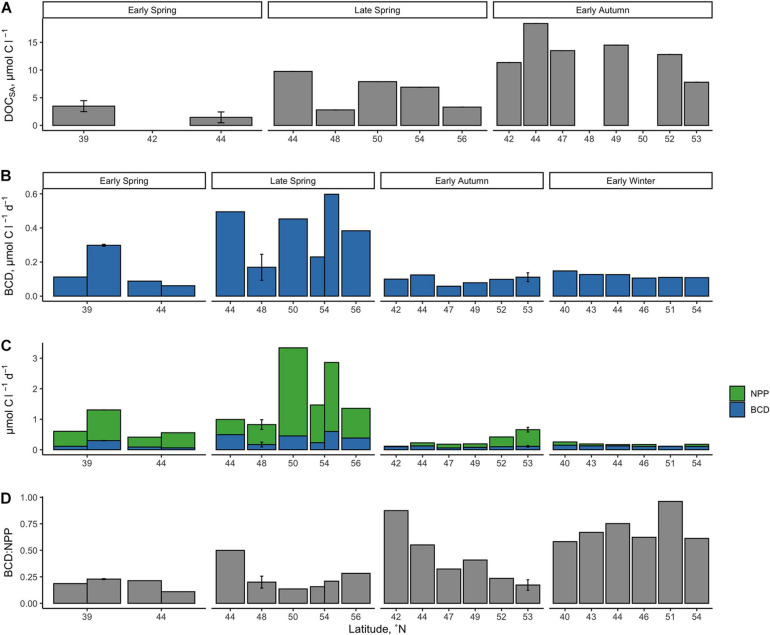
Seasonally accumulated DOC from the initial condition of the DOC remineralization experiments, which were conducted using water from the surface at 10 m **(A)**. BGEs were applied to *in situ* measurements of net bacterio plankton production to estimate bacterioplankton carbon demand [BCD **(B)**] and the fraction of NPP it represents **(C,D)**. Error bars represent standard deviations.

**FIGURE 8:**
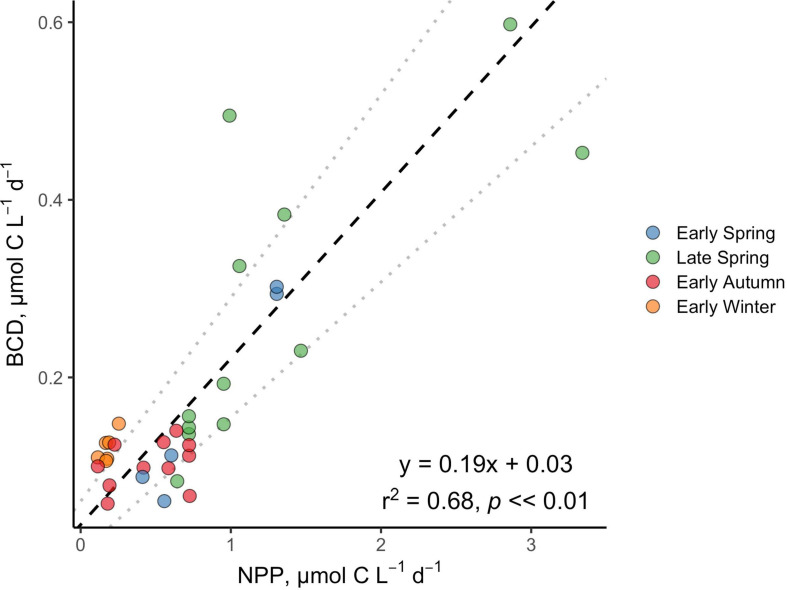
Standardized (reduced) major axis model II linear regression between NPP and BCD. Dotted gray lines indicate the 2.5 and 97.5% confidence intervals.

BCD was calculated for each station by dividing measurements of net BP by the campaign-wide mean BGE of 0.26. The seasonal change in the magnitude of BCD rates exhibited similar patterns as NPP where rates were greatest and most variable in the spring compared to autumn/winter (Table 2 and Figures 7, 8). BCD ranged from 0.06 to 0.60 μmol C l^–1^ d^–1^ and never exceeded NPP (0.11–3.34 μmol C l^–1^ d^–1^). However, BCD:NPP significantly differed between seasons (Kruskal–Wallis *p* < 0.01), being driven by significant decreases from the maximum of 70 ± 14% in early winter to 19 ± 5% in early spring (Wilcoxon *p* = 0.05) and to 22 ± 10% in the late spring (Wilcoxon *p* = 0.01).

## Discussion

This work evaluates both the ecological and biogeochemical contribution of DOM over the progression of a composite annual phytoplankton bloom cycle in the western North Atlantic. Of ecological interest is the instantaneous flux and fate of the most labile constituents of DOM through the bacterioplankton community, whether incorporated into new biomass with potential for trophic transfer or respired into inorganic constituents. Of biogeochemical interest is the portion of DOM that is produced as or transformed into more recalcitrant compounds that can persist and potentially be exported via mixing or subduction. Many previous studies have investigated either the instantaneous flux of DOC (e.g., Teira et al., 2003; Alonso-Sáez et al., 2007) or the seasonal accumulation of a persistent pool of DOC (e.g., Copin-Montégut and Avril, 1993; Carlson et al., 1994; Børsheim and Myklestad, 1997). This study is unique in that it evaluates both fluxes in order to better elucidate and link how heterotrophic bacterioplankton mediate carbon cycling in the NAAMES study region.

### Assessment of BGE in the Western North Atlantic

After DOC is assimilated by bacterioplankton, a fraction is used to fuel anabolism while the rest is catabolized used to generate the ATP necessary for the remaining cellular energetic demands, such as membrane transport, cellular maintenance, and motility. The efficiency by which natural bacterioplankton assemblages repackage DOC into cells and transfer energy to higher trophic levels is partially controlled by BGE (Del Giorgio and Cole, 1998; Carlson and Hansell, 2015). The greater the BGE, the greater the trophic link, the lower the BGE, and the greater the energetic sink within the microbial food web (Ducklow et al., 1986).

Bacterioplankton growth efficiencies used to estimate gross BP are either empirically determined as changes in DOC and cell biomass (e.g., Carlson et al., 1999; Lønborg et al., 2011; Halewood et al., 2012; Wear et al., 2015) or from measures of BP and respiration (e.g., Reinthaler and Herndl, 2005; Alonso-Sáez et al., 2008; Del Giorgio et al., 2011; Lønborg et al., 2011), adopted from the literature (Marañón et al., 2007) or derived from empirical models (Del Giorgio and Cole, 1998; Rivkin and Legendre, 2001; Hoppe et al., 2002). Empirically determined BGE calculations often necessitate the use of carbon conversion factors (CCFs) to express changes in bacterioplankton cell abundance as changes in cell carbon or O_2_ consumption as CO_2_ production. Converting cell abundance to biomass CCFs previously reported for open ocean bacterioplankton range from 5 to >20 fg C cell^–1^ (Lee and Fuhrman, 1987; Fukuda et al., 1998; Gundersen et al., 2002). The calculation of BGE is sensitive to the CCF chosen to estimate cell carbon (Alonso-Sáez et al., 2007), and it can be problematic to apply a single CCF across all data within a DOC remineralization experiment, such as those presented here, as cell sizes can change with cell growth (e.g., Liu et al., 2020; Stephens et al., 2020). Thus, CCFs are a significant source of uncertainty for budgets of ocean carbon flux that rely on constrained estimates of BGEs (Lochte et al., 1993; Burd et al., 2010). Here we circumvent the need for CCFs by directly measuring the change in BOC collected on GF75 (nominal 0.3 μm cutoff, mean cell retention 78 ± 9%) between the initiation of the experiment (*T*_0_) and stationary phase (*T*_*S*__*tationary*_) of growth in each of the DOC remineralization experiments.

The BGEs empirically determined here occupied a small range, with a mean of 26 ± 10% across three different seasons and the broad spatial range of the NAAMES study region (Table 1). These BGEs fall within the range reported for the open ocean (1 to >60%, Del Giorgio and Cole, 1998) and from previous phytoplankton bloom studies (5–62%, (Carlson and Hansell, 2003; Wear et al., 2015). In the Ross Sea, Carlson and Hansell (2003) reported that BGEs increased from ∼5% during the early phase of a phytoplankton bloom to 30–40% in the late stage of phytoplankton bloom senescence, leading the authors to hypothesize that the bioavailable fraction of DOM near the end of the bloom is of a quality that readily meets the metabolic demands of the responding *in situ* bacterioplankton community. Comparatively, BGEs in the southern North Sea were reported to decrease from 25% in the spring and summer to 14% in the fall and 5% in winter (Reinthaler and Herndl, 2005). The authors hypothesized that the corresponding decreases in BGE and NPP was due to a coincident decrease in DOM lability from spring to winter. BGEs in the NW Mediterranean ranged from 3 to 42% and were highest in the winter and spring when chlorophyll *a* concentrations and rates of NPP were elevated, suggesting that relatively high primary productivity was a source of sustained flux of bioavailable DOM (Alonso-Sáez et al., 2008). Wear et al. (2015) also observed a similar relationship between the physiological state of a phytoplankton bloom and BGE variability in the coastal upwelling system of the Santa Barbara Channel (CA, United States). BGEs were observed to be low (min 17%) in early bloom and then increase later (max 62%) as phytoplankton became Si stressed. Over the same time period, *in situ* DOC concentrations, DOC bioavailability, and DOC persistence increased.

In contrast to the previous studies discussed above, our estimates of BGE over the entire study region in the western North Atlantic did not reveal a statistically significant seasonal pattern (i.e., early spring [27 ± 5%], late spring [22 ± 1%], and early autumn [30 ± 15%]; Figure 5 and Table 1). It is possible that the heterogeneity of the physical and chemical environment over the large geographical realm of the NAAMES region obscured linkages between BGE dynamics and phytoplankton bloom stages and their associated processes. In other words, the spatial heterogeneity of the NAAMES region may overwhelm seasonal differences. Thus, seasonality in BGE may have been more pronounced if the empirical determinations were focused on temporal dynamics within narrower geographic regions, each of which may be characterized by differences in DOM availability and bioavailability, community composition (both phytoplankton and bacterioplankton), and nutrient availability. We were not able to resolve significant seasonality in BGE at ∼44°N (21%), the one station occupied in early spring, late spring, and early autumn. Due to logistical limitations, we do not have a similar seasonal DOM remineralization experiments at other latitudinal regions. Thus, there are insufficient data in this study to directly assess seasonal changes in BGEs for other localized regions or mesoscale features (e.g., eddies) of the Western North Atlantic, a topic for future investigation.

### Seasonality in BCD and BCD:NPP Reflect Changes in the Accumulated DOM Pool

Due to the narrow range of empirically derived BGE estimates during the NAAMES cruises, we adopted a universal campaign mean BGE of 26% to estimate BCD. We acknowledge that the application of a universal BGE oversimplifies estimates of BCD and can affect the interpretation of ocean carbon cycling and budgets (Ducklow et al., 2002; Marañón et al., 2007; Burd et al., 2010). Furthermore, our use of a theoretical leucine to CCF to estimate BP also neglects variability in the fate of incorporated leucine in bacterioplankton cells, whether used for biomass production or respiration (del Giorgio et al., 1997). Despite these caveats, we consider our estimates of BCD to be conservative yet realistic of the flux of the most labile DOM required to support gross BP for the NAAMES campaign.

Over the composite annual cycle of the broad NAAMES study region, bacterioplankton abundance, production, and BCD were positively correlated with seasonal variability in NPP (Figures 6, 7B,C, 8 and Table 2). However, while the rates of NPP and BCD were each greatest in the spring, a relatively smaller fraction of NPP was diverted to labile DOM flux compared to autumn, as revealed by the lower BCD to NPP ratio in the spring (Figures 7B,C,D and Table 2). Interestingly, the spatial variability in the scaling of BCD:NPP showed a marked decrease as NPP increased northward in the early autumn (Figures 7C,D and Table 2). This pattern may be due to a general timing phenomenon where blooms peak and decline earlier in southern latitudes than northern latitudes (Bolaños et al., 2021). Living phytoplankton cells can release up to 80% of their primary production as DOM via direct extracellular release, although most studies report that extracellular release ranges between 5 and 20% of NPP (Nagata, 2008; Carlson and Hansell, 2015). If extracellular release truly falls between 5 and 20%, then food web interactions and DOM production processes other than direct phytoplankton release are sources of organic matter that support heterotrophic demand on rapid timescales. Thus, although the organic carbon available for heterotrophic BP was largely constrained by NPP, it is important to recognize that there are many food web processes that result in the production of DOC (Carlson and Hansell, 2015) and volatile organic compounds (Buchan et al., 2014); thus, instantaneous measures of BCD may lag the instantaneous measures of NPP (Billen, 1990; Ducklow et al., 2002).

The greater overall rates of net BP and BCD as well as the elevated bacterioplankton abundances observed in the spring periods indicate that there was a greater flux of labile DOM to bacterioplankton at that time. However, the corresponding low BCD:NPP suggests that a greater fraction of bloom produced organic matter was either partitioned as POM or was exported from the euphotic zone with a smaller fraction accumulating as DOM. During post-bloom periods (autumn/winter), both NPP and BCD rates decreased yet the ratio of BCD:NPP increased, indicating that the flux of the most labile DOC became more strongly coupled to NPP.

### Seasonality in DOC_*SA*_ Bioavailability and Its Implications

The fraction of primary production partitioned as DOC that is not readily available for rapid consumption by the existing heterotrophic community can accumulate and persist over time. The seasonal accumulation of this DOC, termed DOC_*SA*_, represents a portion of NCP (i.e., export production) and, as such, represents the DOC that is potentially available for vertical or horizontal transport (Hansell et al., 1997; Carlson et al., 1998; Hansell and Carlson, 1998). As previously described for the NAAMES study region, the fraction of NCP represented as DOC_*SA*_ increased from late spring to early autumn. This seasonal change in DOC_*SA*_:NCP indicates that a smaller fraction of NCP is partitioned as DOC during the bloom condition (∼11%) and becomes greater under the non-bloom conditions (∼20%) (Baetge et al., 2020).

The successional pattern from bloom to non-bloom states demonstrates that while there was a consistent flux of labile DOM to fuel heterotrophic BP, there were other components of the bulk DOC pool that were produced but remineralized on longer timescales and subsequently accumulated over time. The present study shows that as DOC_*SA*_:NCP increased from the “climax transition” to the “depletion phase” of the phytoplankton bloom cycle in the NAAMES study region (Baetge et al., 2020), the proportion of ΔDOC:DOC_*SA*_ decreased and led to the buildup of a semi-labile DOC pool (Figure 4 and Table 1). From a biogeochemical perspective, it is this semi-labile DOC pool that resists or escapes microbial degradation on short time scales and persists long enough to be mixed or subducted from the epipelagic to the mesopelagic during annual deep convective mixing at some latitudes that represents a DOC export pathway of the biological carbon pump (Copin-Montégut and Avril, 1993; Carlson et al., 1994; Børsheim and Myklestad, 1997; Hansell and Carlson, 2001; Baetge et al., 2020).

The factors that regulate DOC accumulation and its persistence remain elusive in DOM biogeochemistry (Benner and Amon, 2015; Carlson and Hansell, 2015). One hypothesized factor is the “malfunctioning microbial loop” in which heterotrophic DOC consumption is unable to match DOC release due to inorganic nutrient limitation/competition or from predation (Cotner et al., 1997; Thingstad et al., 1997). The production and release of recalcitrant DOM compounds that are intrinsically resistant to heterotrophic utilization by eukaryotes (Aluwihare and Repeta, 1999; Mitra et al., 2014) and prokaryotes (e.g., McCarthy et al., 1998; Kawasaki and Benner, 2006) is another way that DOC can accumulate. The microbial carbon pump posits that as labile DOC compounds are utilized by heterotrophic bacterioplankton, recalcitrant DOM by-products are produced and accumulate (Jiao et al., 2010; Benner and Herndl, 2011). However, DOC may accumulate not just because of its intrinsic resistance to biological uptake and oxidation but also because the “economics” of oxidizing a compound may vary depending on the community structure of the heterotrophic community (Carlson et al., 2009; Treusch et al., 2009; Giovannoni, 2017; Landry et al., 2017; Saw et al., 2020) and the growth factors required to optimize hydrolytic enzyme production or transport regulation (Reintjes et al., 2020; Arnosti et al., 2021). The alternative “molecular diversity hypothesis” proposes that it is not the inherent stability of a DOC compound that results in its accumulation but rather that any one of the millions of DOM compounds is maintained at a concentration too low for a microbe to detect or invest in uptake mechanisms for consequently allowing the compound to accumulate (Arrieta et al., 2015). The vast diversity of DOM molecules may control the accumulation of otherwise bioavailable compounds, precluding any individual molecule from approaching the chemoreceptive threshold of prokaryotes (Kattner et al., 2011), facilitating low encounter rates between substrate and bacteria via molecular diffusion (Stocker, 2012), and/or demanding more energy from heterotrophs to acquire a particular substrate than they may receive from that substrate (thermodynamic inhibition) (LaRowe et al., 2012). Whatever the mechanisms, ΔDOC:DOC_SA_ for the NAAMES campaign decreased from spring to early autumn and resulted in the accumulation of a DOC pool that persisted, potentially becoming available for vertical export during deep winter convective mixing (Baetge et al., 2020) and episodic deep mixing events (Omand et al., 2015; Lacour et al., 2019).

## Conclusion

This study allowed us to resolve the fate of DOM production in the western North Atlantic over various temporal scales. By combining field observations of net BP and DOC variability (Baetge et al., 2020) with DOC remineralization experiments, we demonstrated seasonality in the BCD:NPP ratio as well as changes in the magnitude and bioavailability of the seasonally accumulated DOC pool. On shorter timescales, the flux of the most labile DOC compounds that supported instantaneous BCD rates was greatest in the spring despite a lower BCD:NPP ratio. During periods of low productivity (i.e., early autumn “depletion phase” and early winter “winter transition”), rates of NPP and BCD decreased yet a greater fraction of the daily NPP supported BCD. Our results also demonstrated that during the high-productivity periods (i.e., early spring “accumulation phase” and late spring “climax transition”) of the phytoplankton bloom, a relatively smaller fraction of NCP was partitioned as DOC_*SA*_. However, the DOC that did accumulate had a larger bioavailable fraction than the DOC_*SA*_ present during periods of low productivity.

## Data Availability Statement

The datasets presented in this study can be found in online repositories. The names of the repository/repositories and accession number(s) can be found below: Carlson (2020) Bacterial cell counts and dissolved organic carbon (DOC) measurements from R/V *Atlantis* AT32, AT34, AT38, and AT39-06 in the western North Atlantic Ocean (35°N to 57°N; 45°W) in November 2015, May 2016, September 2017, March/April 2018. Biological and Chemical Oceanography Data Management Office (BCO-DMO). (Version 1) Version Date 2020-09-16 doi: 10.26008/1912/bco-dmo.824623.1.

## Author Contributions

NB and CC conceived the study and experimental design. NB, CC, BS, AN, KM, and KH collected the samples. NB analyzed the data. All authors assisted with the data reduction, contributed to the revision and editing of the final manuscript, aware of and accept responsibility for this manuscript, and have approved the submitted manuscript.

## Conflict of Interest

The authors declare that the research was conducted in the absence of any commercial or financial relationships that could be construed as a potential conflict of interest.

## Footnotes

1 https://upwell.pfeg.noaa.gov/erddap/info/erdMH1chla8day/index.html

2 https://github.com/nbaetge/naames_bioav_ms

3 https://seabass.gsfc.nasa.gov/naames
