# Supplementary material for: The Seasonal Flux and Fate of Dissolved Organic Carbon Through Bacterioplankton in the Western North Atlantic
Source: Front Microbiol. 2021 Jun 18;12:669883. doi: 10.3389/fmicb.2021.669883 (PMC8249739; doi:10.3389/fmicb.2021.669883)
Supplement: Supplementary file 1 [file Data_Sheet_1.pdf]

Supplementary Material

1.1 Positive Pressure Incubation Bottles

5 L Nalgene Biotainer PC bottles were used as the incubation vessels. To sample an incubation, a bottle was pressurized using filtered air, displacing the water volume through a sample line. Bottle caps were modified to accommodate for sampling via positive pressure. Each cap was fitted with two 0.25” (~6.4 mm) Nalgene polypropylene barbed bulkhead fittings, one of which would accommodate a water sample line while the other an air-intake line. A 3.5 mm length of 1/8” ID x 1/4” OD (~3.2- x 6.4 mm) platinum-cured silicon tubing was affixed to the top of each bulkhead fitting.

For the sample line, a 325 mm length of 1/16” ID x 1/8” OD (~0.06- x 3.2-mm) PTFE tubing was passed through one bulkhead fitting to the bottom of the bottle. Another 7.5-mm length of 1/8” ID x 1/4” OD (~3.2- x 6.4-mm) platinum-cured silicon tubing was inserted to the top-end of the PTFE tubing. Inserted between the platinum-cured silicon and PTFE tubing were 1-mm lengths of 1/16” ID x 1/8” OD (~0.06- x 3.2-mm) platinum-cured silicon tubing, which helped to keep the cap air-tight. Attached to the top end of the sample line were both a polypropylene flow-control clamp and a polycarbonate male Luer lock ring x 1/8” (3.2 mm) hose barb adapter. A polycarbonate female Luer lock ring x 1/8” (3.2 mm) hose barb adapter was attached to the second port, which would accommodate the air-intake line.

1.2 Positive Pressure Air Manifold and Sampling

Ambient air was pumped at < 24 kPa using a Fluval Q2 aqurium pump through a Restek refillable hydrocarbon trap. A length of 1/8” ID x 1/4” OD (~3.2- x 6.4 mm) platinum-cured silicon tubing and 1/16” ID x 1/8” OD (~0.06- x 3.2 mm) PTFE tubing was used as the airline. At the end of the airline was a polycarbonate male Luer lock ring x 1/8” (3.2 mm) hose barb adapter, which could be connected to the port of an incubation bottle cap.

Bottles to be sampled were first mixed gently by swirling. After being pressurized with filtered air, ~25 mL of sample is passed through the sample line and discarded. Samples were then collected, in order, for bacterioplankton abundance, total organic carbon (TOC), dissolved

organic carbon (DOC), bacterioplankton carbon, and bacterioplankton community composition. Samples requiring filtration were filtered inline by attaching a filter holder or cartridge directly to the sample line.

When a bottle was not being sampled, its sample line was connected to its air-intake port, sealing the bottle from atmospheric contamination.

1.3 Bacterioplankton Abundances during DOC Remineralization Experiment Setup

Cruise	Station	Whole water	Inoculum (1.2 µm filtrate)	Experimental Mix at T_0
		Cells L^{-1}		
NAAMES 2	1*	$1.84 * 10^9$	$1.68 * 10^9$	$0.46 * 10^9$
NAAMES 2	2	$2.34 * 10^9$	$1.78 * 10^9$	$0.49 * 10^9$
NAAMES 2	3	$1.83 * 10^9$	$1.18 * 10^9$	$0.47 * 10^9$
NAAMES 2	4	$1.89 * 10^9$	$1.31 * 10^9$	$0.25 * 10^9$
NAAMES 2	5*	$1.99 * 10^9$	$1.99 * 10^9$	$0.49 * 10^9$
NAAMES 3	2**	$1.30 * 10^9$	$0.42 * 10^9$	$0.12 * 10^9$
NAAMES 3	3	$1.20 * 10^9$	$0.71 * 10^9$	$0.17 * 10^9$
NAAMES 3	4	$1.13 * 10^9$	$0.62 * 10^9$	$0.23 * 10^9$
NAAMES 3	5*	$1.59 * 10^9$	$1.81 * 10^9$	$0.39 * 10^9$
NAAMES 3	6	$1.81 * 10^9$	$1.41 * 10^9$	$0.42 * 10^9$
NAAMES 4	2RD	$0.65 * 10^9$	$0.51 * 10^9$	$0.15 * 10^9$
NAAMES 4	2RF	$0.78 * 10^9$	$0.52 * 10^9$	$0.15 * 10^9$
NAAMES 4	1	$0.66 * 10^9$	$0.49 * 10^9$	$0.13 * 10^9$
NAAMES 4	2	$1.24 * 10^9$	$0.91 * 10^9$	$0.21 * 10^9$
NAAMES 4	3*	$0.67 * 10^9$	$0.64 * 10^9$	$0.14 * 10^9$
NAAMES 4	4	$0.65 * 10^9$	$0.55 * 10^9$	$0.15 * 10^9$

Supplementary Table 1. Bacterioplankton abundances estimated by epifluorescence microscopy in whole water (*in situ*), inoculum (1.2 µm filtrate), and the 3:7 inoculum: particle free-water experimental mix at the start of each DOC remineralization experiment. On average, 78 ± 16% of the bacterioplankton population in whole seawater passed through the 1.2 µm filter, but in some cases (n = 4, stations marked with "*") was near or above 100% indicative of poor filtration or potentially, errors in the abundance estimates. The whole water bacterioplankton abundance estimate for NAAME 3 Station 2 was anomalous and was not used in calculations. The initial condition of the experiments contained on average 27 ± 5%, suggesting that our dilution was close to the targeted 3:7 ratio of inoculum: particle free-water.

1.4 Long-term Bacterioplankton Abundance in DOC Remineralization Experiments

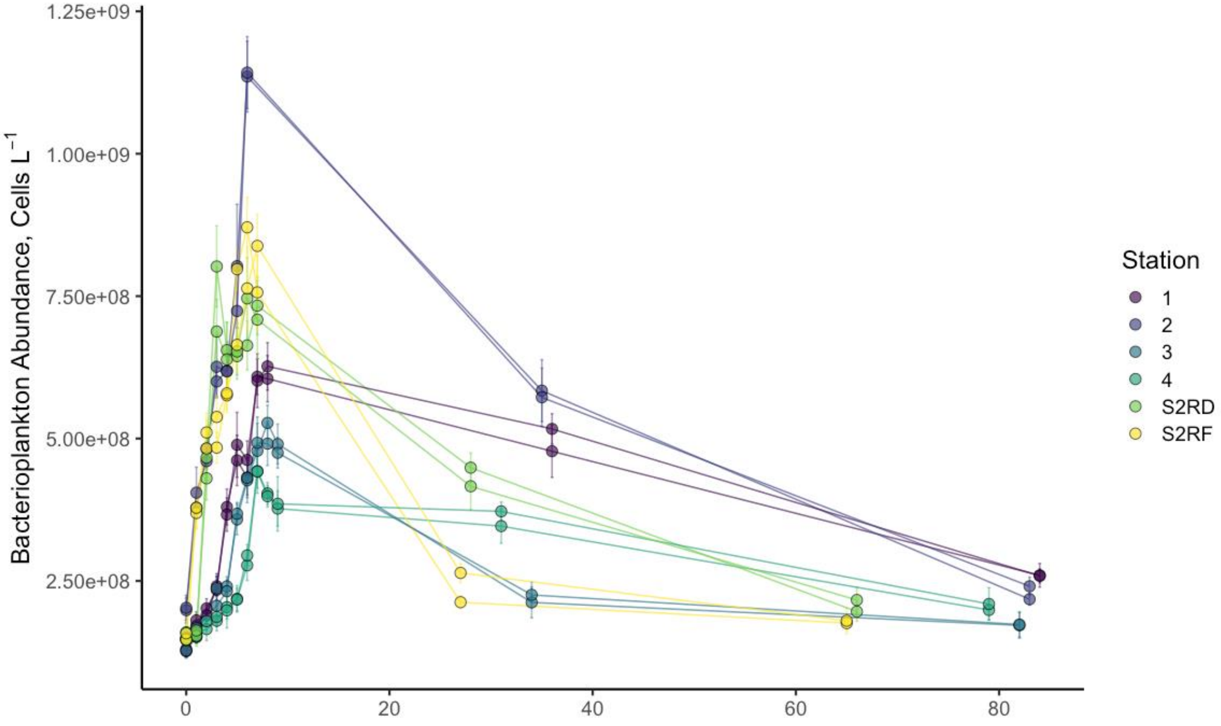

Supplementary Figure 1. Time series of bacterioplankton abundance in DOC remineralization experiments conducted on all stations of NAAMES 4, displaying marked decrease in abundances after 20 days. Data prior to 20 days represent estimates from incubation bottle samples while data at and beyond 20 days represent estimates from incubation vials.

1.5 NAAMES 2 DOC Remineralization Contamination

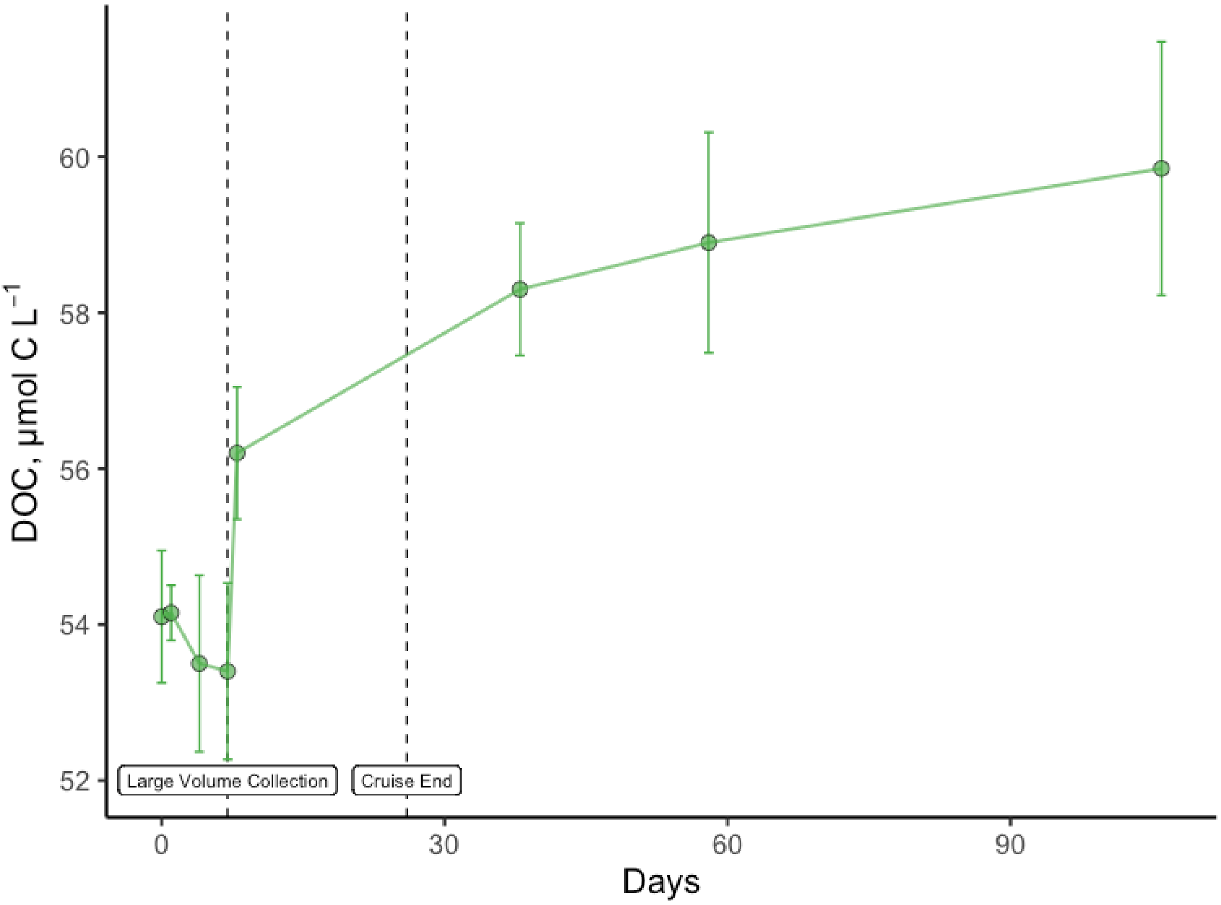

Supplementary Figure 2. DOC remineralization experiment from NAAMES 2 Station 4 displaying marked increase in DOC from day 7 to 8, following removal of large volumes of incubation water for BOC and DNA sampling. The day after drawing large volume samples, DOC increased 2.8 µmol C L^{-1} (5.2%) and continued to increase throughout the post-cruise shore-based incubation and sampling period (98 days), casting uncertainty in our ability to observe long-term trends after removing large volumes of incubation water.

1.6 DOC versus DOC* Comparison

A direct comparison between filtered DOC concentrations and corresponding DOC* estimates from experiments conducted on both NAAMES 3 and 4 showed that the data were significantly and highly correlated. Filtered DOC concentrations were on average 1.6 µmol C L^{-1} higher than DOC*, a difference near the threshold of analytical resolution, with 95% limits of agreement at 4.1 and -0.9 µmol C L^{-1}. While the stacked GF75 filters retained 78 ± 9% of cells on average, the remainder that passes through the filters may account for some of the systematic positive bias of the filtered DOC measurements relative to DOC*. Because of the observed agreement between the incubation container measurements as well as the availability of data, the present analyses used filtered DOC for NAAMES 2 experiments DOC* for NAAMES 3 and 4 experiments to maximize the use of available data and minimize measurement error due to sample handling.

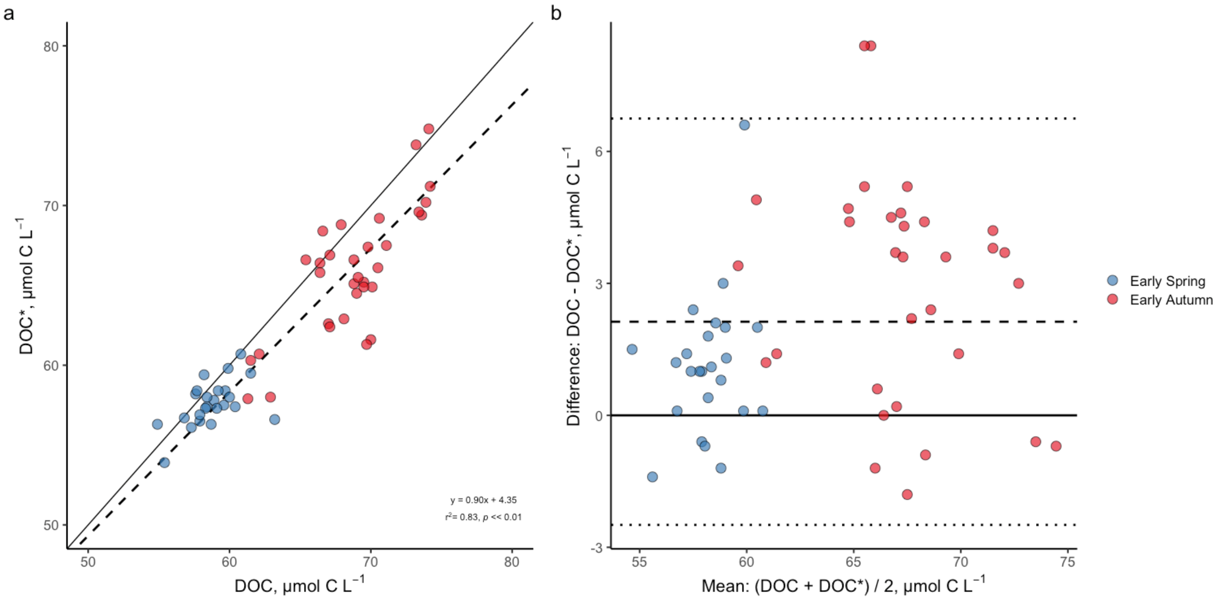

Supplementary Figure 3. Standardized (reduced) major axis model II linear regression (a) and Bland-Altman/Tukey Mean-Difference plot (b) between DOC concentrations measured filtered samples and estimated by subtracting estimates of bacterioplankton biomass (i.e. BOC) from corresponding measurements of TOC (DOC*). Solid and dashed lines in panels a represents the identity and regression lines, respectively. In panel b, the solid line represents the no-bias line, the dashed line represents the mean-difference or bias line, and the dotted lines represent the 95% limits of agreement.

1.7 Incubation Vessel Comparison

Single samples for bacterioplankton abundance and replicate DOC samples were drawn over time from both incubation containers over several experiments during NAAMES 4. Measured bacterioplankton abundances and filtered DOC concentrations between the vessel incubations were highly and significantly correlated, with a slight bias towards bottle incubation measurements which were higher by 2.4 x 10^6 cells L^{-1} and 0.39 µmol C L^{-1} for bacterioplankton abundance and DOC, respectively. The 95% limits of agreement were at 3.4 x 10^8 and -3.4 x 10^8 cells L^{-1} for the bacterioplankton abundance measurements and at 2.6 and -1.8 µmol C L^{-1} for the DOC measurements, indicating that both incubation containers tracked similar microbial dynamics.

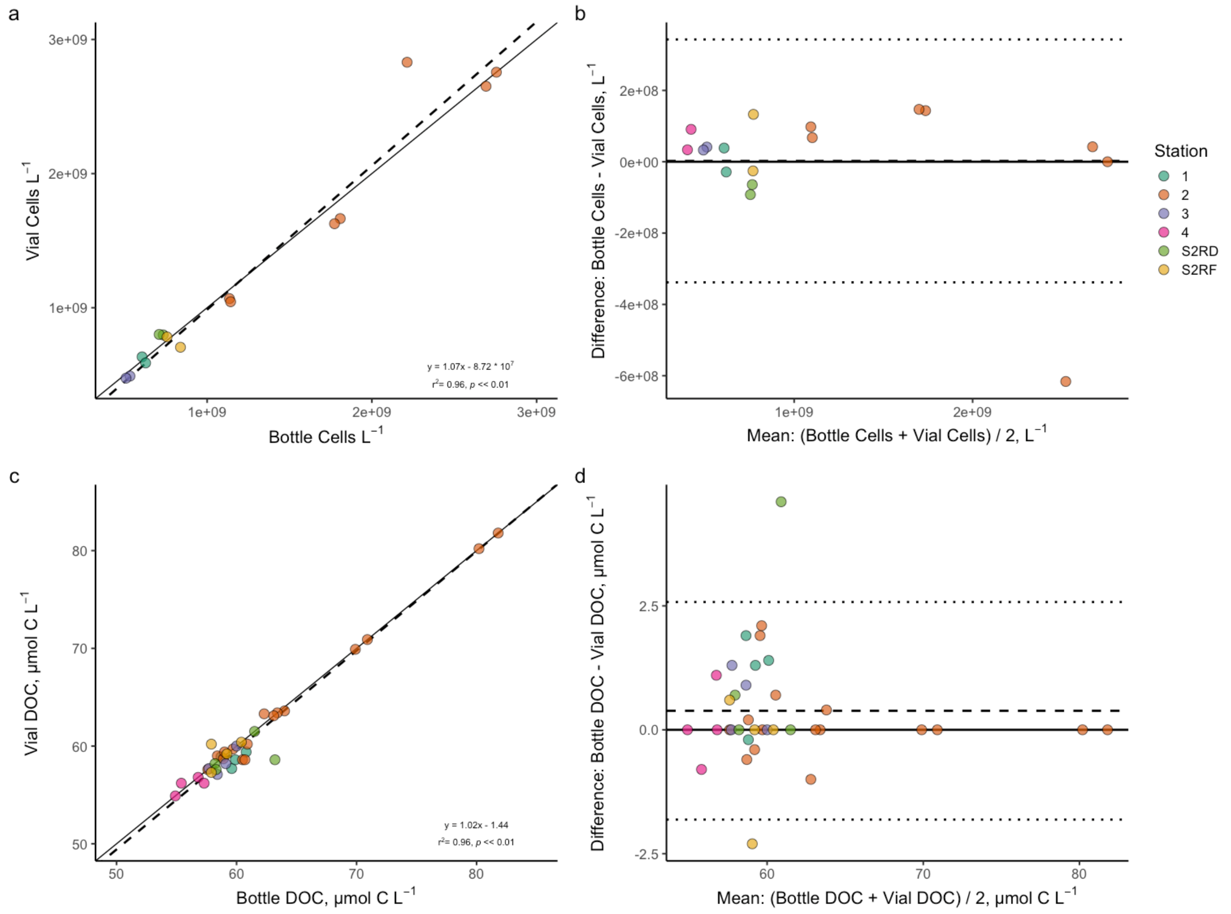

Supplementary Figure 4. Standardized (reduced) major axis model II linear regression (a, c) and Bland-Altman/Tukey Mean-Difference plots (c, d) between cell abundances (a, b) and DOC concentrations (c, d) measured from bottle and vial incubations conducted on NAAMES 4 (early spring). Solid and dashed lines in panels a and c represent the identity and regression lines, respectively. In panels b and d, the solid line represents the no-bias line, the dashed line

represents the mean-difference or bias line, and the dotted lines represent the 95% limits of agreement.
